# Hospitalization Causes and Epidemiological Characteristics Among Patients With End-Stage Renal Disease: A Six-Year Retrospective Study

**DOI:** 10.7759/cureus.66582

**Published:** 2024-08-10

**Authors:** José Manuel García Romero, Raúl Melo Acevedo, José Ignacio Mercado Merino, Fatima Paulina Jaime Vargas, Nemi Isabel Pérez Peña, Francisco Ortega Arreola, Ana Laura Alegria Arias, José Gonzalo Bravo Quiroz, Pablo Hernández Guillén, Luis Fernando Torres Monroy

**Affiliations:** 1 Transplant and Donation, Regional General Hospital 1 of the Mexican Social Security Institute, Querétaro, MEX; 2 Internal Medicine, Regional General Hospital 1 of the Mexican Social Security Institute, Querétaro, MEX; 3 General Practice, Autonomous University of Querétaro, Querétaro, MEX; 4 General Practice, Health Clinic 56 Amealco of the Mexican Social Security Institute, Querétaro, MEX; 5 General Practice, Centro de Salud Pedro Escobedo, Secretaría de Salud, Querétaro, MEX; 6 General Practice, Universidad Anáhuac México, Mexico City, MEX

**Keywords:** peritoneal dialysis (pd), hemodialysis, chronic kidney disease, epidemiology, renal dialysis, hospitalization, renal insufficiency

## Abstract

Background: Chronic kidney disease (CKD) leads to a high rate of complications requiring hospital admission for advanced management. Therefore, this study aims to analyze the main causes of hospitalization following the initiation of renal replacement therapy (RRT).

Materials and methods: This observational and descriptive study utilized a non-probabilistic quota sampling method, reviewing a total of 423 medical records from General Regional Hospital 1 of the Mexican Social Security Institute in Querétaro. The study evaluated the frequency and causality of hospitalizations during a retrospective period from 2018 to 2023.

Results: There were 1,162 hospitalization events involving 423 patients; 71.63% of patients started RRT with peritoneal dialysis, while 26% began with hemodialysis. The leading cause of hospitalization was electrolyte imbalance (397; 34.17%), followed by peritonitis associated with peritoneal dialysis (351; 30.21%), change to hemodialysis (270; 23.24%), Tenckhoff catheter dysfunction (209; 17.99%), and fluid overload (205; 17.64%). The group with the highest number of events was renal-related complications, followed by infectious causes.

Conclusions: Hospitalizations in end-stage CKD patients often arise from the complex renal pathophysiology and complications related to acute and decompensated renal function. This condition refers to the kidneys' failure to maintain essential physiological functions despite ongoing treatment, leading to issues such as electrolyte imbalances, fluid overload, and uremic syndrome. To reduce morbidity and mortality, measures such as enhanced training in ambulatory dialysis, improved catheter care, and early infection detection are crucial. A comprehensive approach that addresses both acute issues and preventive strategies is essential for improving clinical outcomes and quality of life for these patients.

## Introduction

Chronic kidney disease (CKD) is a significant global and national health issue, affecting approximately 9.1% of the global adult population, with diabetes mellitus and hypertension as the primary drivers [1,2]. Diabetes is a major contributor to CKD, leading to nephron damage and a decline in the glomerular filtration rate. The global burden of CKD also encompasses challenges in health-related quality of life and employment, particularly for patients undergoing dialysis [3,4].

In Mexico, CKD prevalence is notably high, affecting approximately 12-15% of the adult population, which is significantly higher than the global average. This elevated prevalence is largely due to high rates of diabetes mellitus and hypertension, which are the primary contributors to CKD in the country. The high prevalence rates create a substantial demand for healthcare resources. The economic burden is further compounded by the need for frequent medical interventions and hospitalizations, which place additional stress on the healthcare infrastructure [5].

Consequently, when patients progress to end-stage renal disease (ESRD), the implementation of renal replacement therapies (RRT), such as hemodialysis, peritoneal dialysis, or kidney transplantation, becomes essential [6]. Although these therapies extend the lives of patients with kidney disease, they also introduce additional comorbidities and associated complications [7].

The selection of management modality for ESRD depends on several factors, including age, gender, presence of comorbidities, life expectancy, and support network, with kidney transplantation being the preferred choice [8]. However, due to the high demand for kidney grafts and limited organ availability, patients with ESRD often require alternative therapies while awaiting transplantation, whether from a living or deceased donor [9].

Monitoring and maintaining patients undergoing RRT present a substantial challenge for public health due to numerous complications that elevate morbidity and mortality [10,11]. Individuals with ESRD experience frequent hospital admissions due to a broad spectrum of complications linked to the disease's pathophysiology and the inherent complications of RRT [12]. The complexities associated with CKD necessitate comprehensive multidisciplinary management due to its multisystem impact, intricate treatment regimens, and significant psychosocial effects. Managing CKD involves addressing cardiovascular issues, bone mineral disorders, and fluid imbalances, along with supporting patient self-management. A holistic approach that includes ethical, emotional, and palliative care considerations is crucial for improving patient outcomes and quality of life [8].

Cardiovascular disease stands as one of the leading causes of mortality, particularly affecting patients with ESRD, who face elevated cardiovascular risks [13]. The risk factors associated with the development of ESRD, such as obesity, hypertension, diabetes mellitus, smoking, and male gender, contribute significantly to this heightened cardiovascular risk [14]. Go et al. found that CKD is associated with increased risks of death, cardiovascular events, and hospitalizations, with advanced stages of the disease leading to higher mortality rates and more severe complications [12].

The quality of life for dialysis patients is notably reduced due to various physical and emotional challenges. Brown et al. reported that both peritoneal dialysis and hemodialysis patients experience diminished health-related quality of life, with issues arising from symptoms, activity limitations, and psychological stress [3]. Additionally, Davies et al. highlighted that long-term peritoneal dialysis patients often face frequent hospitalizations due to complications such as infections, emphasizing the need for comprehensive care to effectively address these challenges [15]. Therefore, analyzing the primary causes of hospitalization in this patient population is not only feasible but also crucial for guiding preventive measures and enhancing medical care. Such efforts can potentially lead to tangible improvements in quality of life and reductions in morbidity and mortality rates among patients undergoing RRT for ESRD.

## Materials and methods

**Figure 1:**
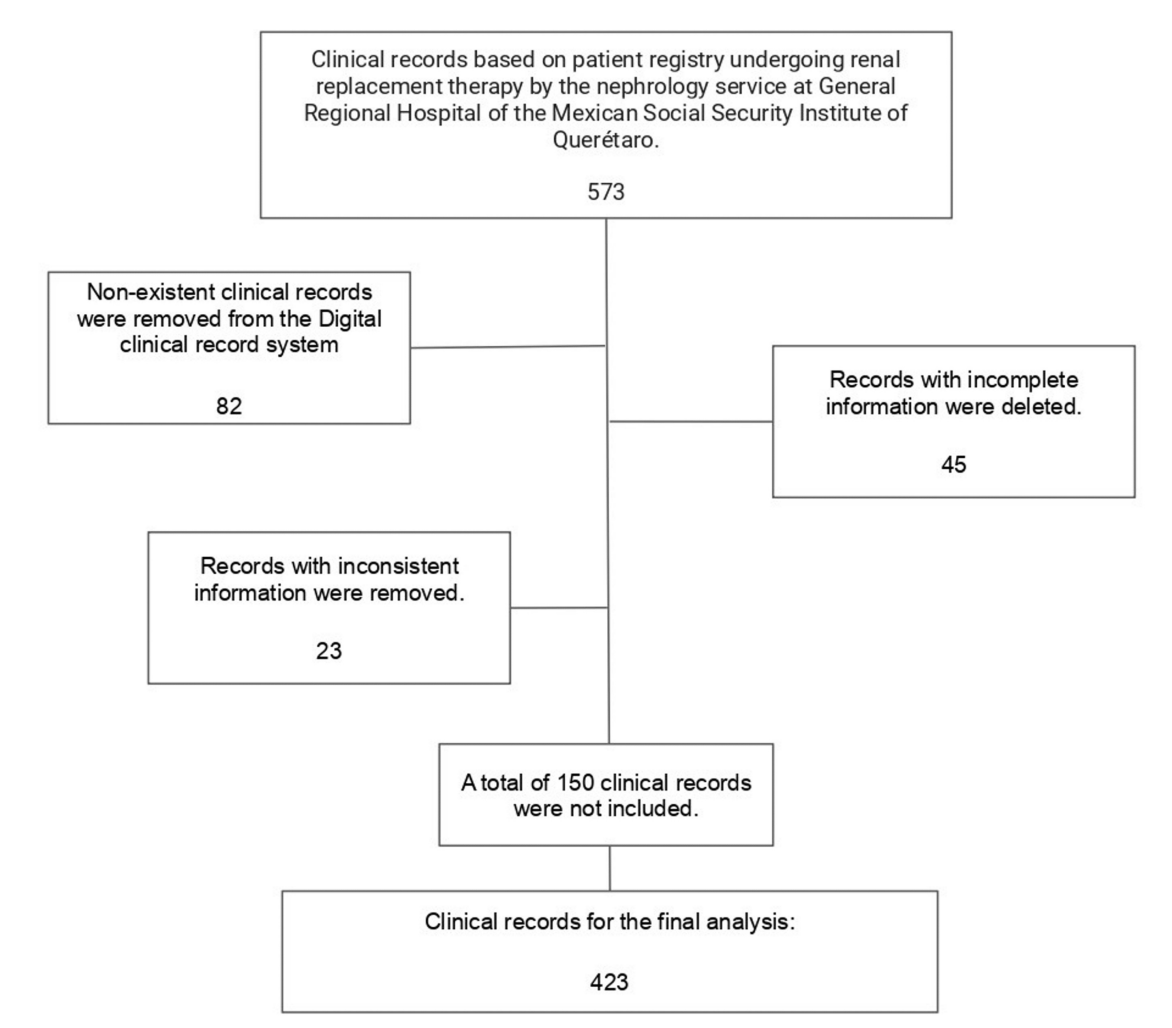
Flowchart of patient selection with ESRD from January 2018 to October 2023 The process began with 573 records, which were subjected to inclusion and exclusion criteria, resulting in 423 records remaining. ESRD: end-stage renal disease

In this retrospective study, we analyzed the electronic medical records of patients diagnosed with CKD, according to the International Classification of Diseases, Eleventh Revision, who were undergoing some form of RRT at General Regional Hospital 1 of the Mexican Social Security Institute in Querétaro. The study encompassed records from January 2018 to December 2023. Inclusion criteria required patients to be classified as CKD stage V based on the KDIGO guidelines at or before the time of hospitalization and to have a complete medical record. After applying these criteria to an initial pool of 573 clinical files, 423 records were selected for inclusion using a non-probabilistic quota sampling method (Figure 1).

To determine the appropriate sample size for this study, we utilized Cochran’s formula, a widely recognized method for calculating sample sizes in epidemiological studies [16]. This formula helped ensure that the sample was statistically adequate for analyzing the causes of hospitalization and assessing associated outcomes.

Causes of hospitalization were categorized as non-mutually exclusive, which allowed for the documentation of multiple causes per patient, if applicable. This approach ensured that all relevant data regarding each patient’s hospitalization was captured comprehensively. Additionally, the Charlson Comorbidity Index was calculated to evaluate the burden of comorbid conditions and estimate mortality risk within the study population. Mortality rates were stratified based on the index scores: a score of 0 was associated with a 12% mortality rate, scores of 1-2 with a 26% mortality rate, scores of 3-4 with a 52% mortality rate, and a score ≥5 with an 85% mortality rate [17].

Ethical approval for the study was granted by the Local Health Research Committee 2201 and the Research Ethics Committee 22018, with approval facilitated through the Institutional Platform of the Mexican Social Security Institute Electronic Research Record System (approval number: R-2024-2201-050). Given the retrospective design and minimal risk associated with the study, the institutional review board exempted the need for informed consent from patients, as the study primarily involved the analysis of existing medical records.

Statistical analysis

Data were collected over a four-month period and included the following variables: age, sex, comorbidities, etiology of renal disease, type of RRT first initiated, and causes of hospitalization over a six-year period. Statistical analyses were conducted using SPSS Statistics version 25.0 (IBM Corp. Released 2017. IBM SPSS Statistics for Windows, Version 25.0. Armonk, NY: IBM Corp.). A descriptive statistical approach was employed to summarize the data, which included calculating percentages for categorical variables and means ± standard deviations (SD) or medians (IQR) for continuous variables, as appropriate for the data distribution.

## Results

**Figure 2:**
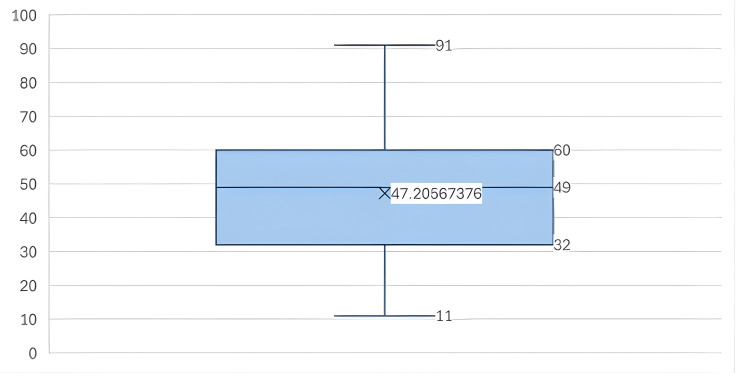
Age distribution among patients with end-stage CKD under RRT Box plot showing ages with a mean of 47.21 (±15.58), median of 49 (32-60), and minimum and maximum age values of 11 and 91 years, respectively. CKD: chronic kidney disease, RRT: renal replacement therapy

During the study period, a total of 1162 hospitalization events were evaluated, involving 423 patients undergoing some form of RRT. Among these patients, 337 (79.67%) experienced between one and four hospitalizations, while only 15 (3.55%) did not have any hospitalization events. The majority of patients were male (60.28%). The median age at diagnosis was 49 years (IQR 32-60) (Figure 2).

**Figure 3:**
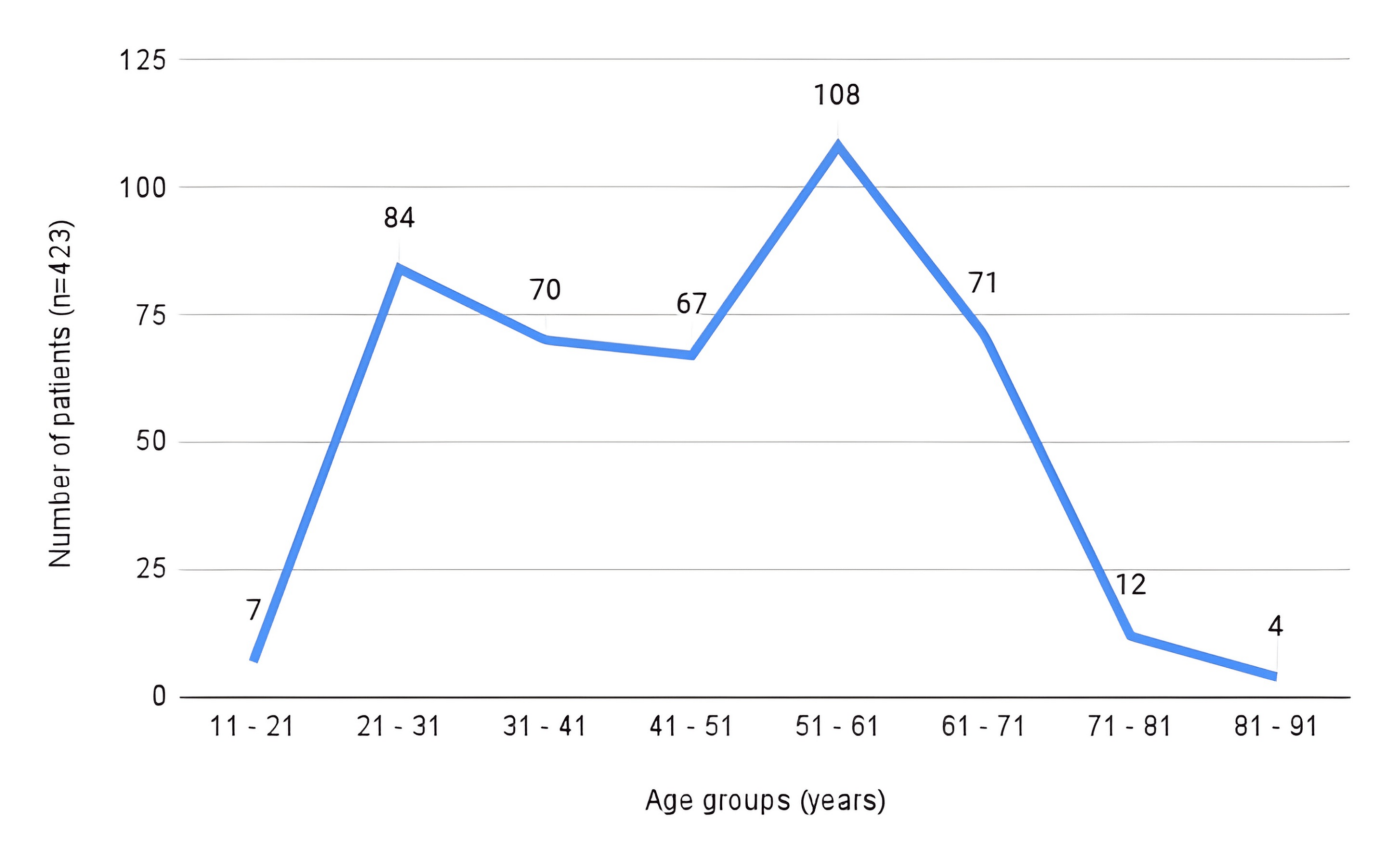
Age groups within the study population Eight age groups are depicted, showing the distribution within the study group marked in completed years.

Two predominant age groups were identified: the primary group consisted of individuals aged 51-61 years (108; 25.53%), followed by the age group of 21-31 years (84; 19.85%) (Figure 3).

The most frequent comorbidities observed were anemia (92.91%), hypertension (75.41%), type 2 diabetes (45.15%), and secondary hypertension (15.37%). The primary causes of nephropathy identified were hypertensive nephropathy and diabetic nephropathy (39.72%), along with isolated hypertensive nephropathy (23.17%) and renal hypoplasia (17.02%). Although renal biopsies were not performed on all patients due to resource limitations and high demand for chronic patient care, the diagnosis of hypertensive and diabetic nephropathy was based on a combination of clinical, laboratory, and, in some cases, imaging evidence. Patients with diabetic nephropathy typically presented with persistent proteinuria and worsening renal function associated with diabetes, while those with hypertensive nephropathy showed signs of renal damage related to chronic hypertension. Ultrasound findings, although not specific, supported the diagnosis by revealing structural changes in the kidneys. Given the lack of definitive biopsy confirmation, the diagnosis relied on available clinical and laboratory evidence, which strongly indicated hypertension and diabetes as the primary causes of renal damage in the studied population.

In terms of initial RRT, 71.63% of patients began with peritoneal dialysis, while 26% started with hemodialysis. The mean Charlson Comorbidity Index was 2.8 (SD 0.89), ranging from 2 to 6 (Table 1).

**Table 1:** Demographic data, etiology of nephropathy, and comorbidities of the study group

				n (%)
Demographic characteristics	Male gender			255 (60.28)
	Female gender			168 (39.72)
Cause of nephropathy	Hypertensive + diabetic etiology			168 (39.72)
	Hypertensive etiology			98 (23.17)
	Renal hypoplasia etiology			72 (17.02)
Comorbidities	Anemia			393 (92.91)
	Hypertension			319 (75.41)
	Type 2 diabetes			191 (45.15)
	Secondary hypertension			65 (15.37)
	Hypothyroidism			34 (8.04)
	Peritoneal dialysis as initial therapy			303 (71.63)
	Hemodialysis as initial therapy			110 (26)
	Renal transplantation as initial therapy			10 (2.36)

Of the 1162 hospitalizations analyzed, the primary causes were electrolyte imbalance, encompassing hyperkalemia, hypocalcemia, hyperphosphatemia, hypomagnesemia, and metabolic acidosis (397; 34.17%), secondary bacterial peritonitis associated with peritoneal dialysis (351; 30.21%), change to hemodialysis (270; 23.24%), Tenckhoff catheter dysfunction, including catheter migration, inflow failure, outflow failure, fibrin clot, and accidental removal (209; 17.99%), and fluid overload (205; 17.64%).

Causes of hospital admission related to acute decompensated CKD represented the largest group of hospitalizations, comprising 714 out of 1162 events (61.45%). In the context of RRT, this condition reflects the kidneys' failure to sustain essential physiological functions despite ongoing treatment. It signifies an inability to effectively manage metabolic and fluid balance, even with support from dialysis or other RRT. The most frequent complications within this group were electrolyte imbalance (397; 34.17%), fluid overload (205; 17.64%), and uremic syndrome (112; 9.64%).

**Figure 4:**
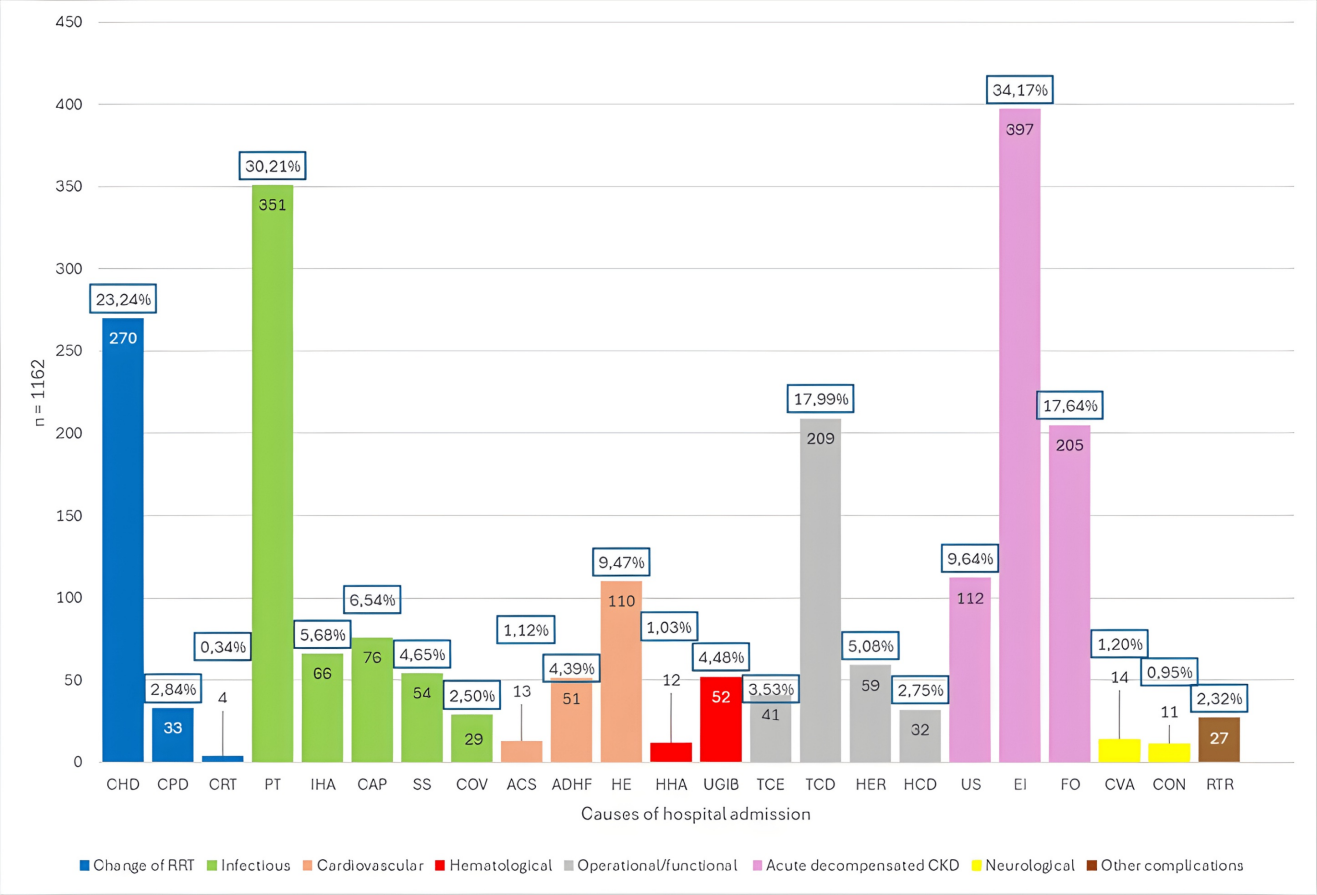
Number of hospital admissions by etiology groups over six years (n=1162) The figure displays the number of events for each cause of hospitalization along with its percentage of total admissions (n=1162). CKD: chronic kidney disease, RRT: renal replacement therapy, CHD: change to hemodialysis, CPD: change to peritoneal dialysis, CRT: change to renal transplantation, PT: peritonitis, IHA: infection of the hemodialysis access, CAP: community-acquired pneumonia, SS: septic shock, COV: COVID-19, ACS: acute coronary syndrome, ADHF: acute decompensated heart failure, HE: hypertensive emergency, HHA: hemorrhage from hemodialysis access, UGIB: upper gastrointestinal bleeding, TCE: Tenckhoff catheter exchange, TCD: Tenckhoff catheter dysfunction, HER: abdominal/inguinal hernia, HCD: hemodialysis catheter dysfunction, US: uremic syndrome, EI: electrolyte imbalance, FO: fluid overload, CVA: cerebrovascular accident, CON: convulsions/seizures, RTR: renal transplant rejection

The second-highest group of hospitalizations was due to infectious causes, totaling 576 out of 1162 events (49.56%). This category encompassed peritonitis associated with peritoneal dialysis (351; 30.21%), community-acquired pneumonia (76; 6.54%), hemodialysis access infection (66; 5.68%), septic shock (54; 4.56%), and COVID-19 (29; 2.50%) (Figure 4).

Of the 66 hospitalization events due to hemodialysis access infections, 65% were due to infections of the Permacath catheter, 30% were due to infections of the Mahurkar catheter, and 5% were due to infections of the AV fistula. Four primary causes demonstrated a high recurrence rate among patients with ESRD: peritonitis associated with peritoneal dialysis (1.9), electrolyte imbalance (1.6), uremic syndrome (1.6), and hypertensive emergency (1.5) (Table 2).

**Table 2:** Number of patients with hospital admissions and number of hospitalizations by cause The number of hospitalizations is shown for each cause of hospitalization as well as the percentage of total admissions (n=1162). To determine the frequency of events (rate) relative to the number of individuals, divide the total number of hospital admissions by the number of patients who experienced that event.

		Number of patients (n=423)	Total hospital admissions (n= 1162)	Rate (2.7)
Change in RRT	Change to hemodialysis	253	270	1.1
	Change to peritoneal dialysis	33	33	1.0
	Change to renal transplant	4	4	1.0
Infectious cause	Peritonitis	182	351	1.9
	Infection of hemodialysis access	55	66	1.2
	Community-acquired pneumonia	65	76	1.2
	Septic shock	50	54	1.1
	COVID-19	28	29	1.0
Cardiovascular cause	Acute coronary syndrome	10	13	1.3
	Acute decompensated heart failure	39	51	1.3
	Hypertensive emergency	74	110	1.5
Hematological cause	Hemorrhage from hemodialysis access	11	12	1.1
	Upper gastrointestinal bleeding	44	52	1.2
Operational/functional cause	Tenckhoff catheter exchange	38	41	1.1
	Tenckhoff catheter dysfunction	163	209	1.3
	Abdominal/inguinal hernia	51	59	1.2
	Hemodialysis catheter dysfunction	26	32	1.2
Acute decompensated CKD	Uremic syndrome	105	112	1.1
	Electrolyte imbalance	254	397	1.6
	Fluid overload	131	205	1.6
Neurological cause	Cerebrovascular accident	13	14	1.1
	Convulsions/seizures	11	11	1.0
Other complications	Renal transplant rejection	26	27	1.0
	Death	11	11	1.0

These findings underscore the complexity and severity of complications experienced by these patients, significantly impacting their quality of life and contributing to increased morbidity and mortality.

## Discussion

This study investigated the leading causes of hospitalization in patients with ESRD, covering various treatment modalities, including peritoneal dialysis, hemodialysis, and kidney transplantation. Our findings underscored that the predominant primary nephropathies were closely linked to chronic conditions such as hypertension and diabetes mellitus, aligning with established literature on risk factors contributing to the development of ESRD [4].

Regarding age group distribution, our study identified two significant cohorts: the 21-31 year group and the 51-61 year group (Figure 3). It is hypothesized that the first cohort may be more affected by congenital nephropathies such as renal hypoplasia and renal agenesis, while the second cohort demonstrated a higher prevalence of chronic diseases like hypertension and diabetes, which aligns with previous research in this area [2].

In terms of the type of RRT initiation, our findings indicated a marked trend towards the use of peritoneal dialysis. This preference can be attributed to its ambulatory nature and lower cost compared to other modalities. In Mexico, peritoneal dialysis is preferred due to its cost-effectiveness, significant government support, and logistical advantages. It is less expensive than hemodialysis because it requires fewer resources and staff, making it financially accessible. It also offers logistical benefits by allowing patients to perform the treatment at home, thus reducing the need for frequent travel to dialysis centers. These combined factors make peritoneal dialysis a feasible and advantageous option for RRT in Mexico [18,19]. However, as reported by Argaiz et al., CKD in Mexico carries a high burden of mortality, so identifying and treating complications such as peritoneal dialysis-associated peritonitis early would improve survival rates and have a direct impact on patients' quality of life and mortality [5].

According to Schrauben et al., the most common causes of hospitalization among American ESRD patients were predominantly related to cardiovascular issues, highlighting the significant impact of cardiovascular complications in this population [20]. However, our study reveals a different pattern, where the primary causes of hospitalization were associated with acute decompensated CKD. Specifically, the leading causes included electrolyte imbalance (397; 34.17%), fluid overload presented as pulmonary edema, ascites, weight gain, and edema (205; 17.64%), secondary bacterial peritonitis associated with peritoneal dialysis (351; 30.21%), and RRT dysfunctions, including changes to hemodialysis (270; 23.24%) and Tenckhoff catheter dysfunction (209; 17.99%).

This disparity may reflect regional or population-specific differences in the prevalence and management of ESRD complications. While cardiovascular issues remain a critical concern and are prominent in the American ESRD population, our findings underscore the substantial burden of renal-related complications and infections in our study group. Furthermore, the differences in findings may be attributed to variations in healthcare practices, patient demographics, and regional healthcare infrastructure. It is important to highlight that, although cardiovascular causes were not the most frequent in our study, the impact of cardiovascular issues on increased mortality remains significant [13]. The high rates of electrolyte imbalances and fluid overload suggest that, even with advanced RRT, patients face considerable challenges that necessitate targeted interventions. Therefore, both cardiovascular factors and those related to renal function must be addressed to improve overall patient outcomes and reduce hospitalization rates.

This study demonstrated a high number and recurrence of peritonitis cases associated with peritoneal dialysis (351; 30.21%). These results are consistent with those reported by Piraino and Sheth, who identified peritonitis not only as a significant complication related to peritoneal dialysis but also as a major risk factor for death in CKD patients [21].

The literature indicates that infection of hemodialysis access is a cause of high morbidity and mortality in patients with CKD. However, in our results (66 cases; 5.68%), as reported by Schwanke et al., the frequency of infection of hemodialysis access was found to be less than 10% of cases [22].

Compared to what was reported in the study by Metcalfe et al., the frequency of changes to hemodialysis (270; 23.24%), peritonitis (351; 30.21%), and fluid overload (205; 17.64%) was higher in our study. However, the frequency of hemodialysis access infections in our study was lower (66; 5.68%). This highlights the need for measures to reduce the frequency of complications that directly impact the patient's quality of life as well as the critical role of renal function management in hospital admissions [23].

Peritonitis associated with peritoneal dialysis accounted for a substantial portion of hospitalization events (30.21%), along with Tenckhoff catheter dysfunction (17.99%). These findings suggest that complications specific to peritoneal dialysis patients contribute significantly to increased morbidity and mortality within this group undergoing RRT [24-26].

Additionally, 29% of the 423 patients evaluated exhibited cardiovascular compromise, including hypertensive crisis, acute coronary syndrome, and/or acute decompensated heart failure. This observation aligns with existing literature highlighting the heightened cardiovascular risk in individuals with CKD. Hypertension and diabetes mellitus are primary contributors, with both conditions exacerbating cardiovascular risk through mechanisms such as endothelial dysfunction, increased arterial stiffness, atherosclerosis, dyslipidemia, and chronic inflammation, which further accelerate cardiovascular issues by promoting plaque buildup and endothelial damage [20,27]. Additionally, anemia, often present in CKD, strains the heart and contributes to left ventricular hypertrophy and heart failure, while secondary hyperparathyroidism and mineral bone disorders increase vascular calcification and stiffness [28,29].

Other factors, such as obesity, smoking, and a sedentary lifestyle, compound the risk of worsening hypertension, diabetes, and systemic inflammation. Advanced age, genetic predispositions, and family history of cardiovascular disease also play a role, as do dialysis-related stress and complications in patients undergoing RRT. Addressing these multifaceted risk factors through lifestyle changes, targeted treatments, and regular monitoring is essential to mitigate cardiovascular risk and enhance overall health in the CKD population [4].

Furthermore, the study found a mean Charlson Comorbidity Index of 2.8, which correlates with a projected mortality rate ranging from 26% to 52%. In the study conducted by Mix et al., it was established that morbidity and mortality in patients increase as they progress to ESRD. Additionally, a significant percentage of patients have an increased cardiovascular risk. Therefore, identifying the causes of hospitalization could improve clinical outcomes by focusing on the prevention and proper management of cardiovascular, renal, and infectious complications in ESRD patients [30,31].

It is important to mention the limitations of our study, such as the exclusion of hospital stay duration as a study variable and the lack of quantitative variables like glomerular filtration rate and creatinine levels. Additionally, it may be useful to separate the causes of hospitalization by age group, gender, or type of RRT to conclude the frequency of such hospitalizations in each group. For future research, considering these factors could help identify opportunities to improve the prevention of complications and optimize the management of hospital care for patients with ESRD.

## Conclusions

The primary causes of hospitalization in patients with ESRD underscore the intricate nature of renal pathophysiology, where the kidneys' central role leads to diverse manifestations and complications. However, it's essential to note that infectious diseases also play a significant role in hospital admissions. Implementing effective measures to mitigate the risk of infections, such as providing comprehensive training in ambulatory dialysis, enhancing catheter care and placement techniques, and early identification of infections, could potentially reduce morbidity and mortality in this patient group.

Addressing complications stemming from ESRD, whether intrinsic to the condition or associated with therapeutic procedures, is paramount for enhancing clinical outcomes and patient well-being. This requires a comprehensive approach that not only manages acute medical issues effectively but also prioritizes preventive strategies. By optimizing care and mitigating the causes of hospitalization, we can significantly improve the long-term quality of life for these patients.
